# Ecological release in White Sands lizards

**DOI:** 10.1002/ece3.50

**Published:** 2011-12

**Authors:** S Des Roches, J M Robertson, L J Harmon, E B Rosenblum

**Affiliations:** 1 Department of Biological Sciences, University of IdahoMoscow; 2 Department of Biological Sciences, Colorado State UniversityFort Collins, Colorado

**Keywords:** Adaptation, colonization, density compensation, ecological opportunity, ecological release, natural selection, reptiles, selection, speciation, White Sands

## Abstract

*Ecological opportunity* is any change that allows populations to escape selection from competition and predation. After encountering ecological opportunity, populations may experience *ecological release*: enlarged population size, broadened resource use, and/or increased morphological variation. We identified ecological opportunity and tested for ecological release in three lizard colonists of White Sands, New Mexico (*Sceloporus undulatus*, *Holbrookia maculata*, and *Aspidoscelis inornata*). First, we provide evidence for ecological opportunity by demonstrating reduced species richness and abundance of potential competitors and predators at White Sands relative to nearby dark soils habitats. Second, we characterize ecological release at White Sands by demonstrating density compensation in the three White Sands lizard species and expanded resource use in White Sands *S. undulatus*. Contrary to predictions from ecological release models, we observed directional trait change but not increased trait variation in *S. undulatus*. Our results suggest that ecological opportunity and ecological release can be identified in natural populations, especially those that have recently colonized isolated ecosystems.

## Introduction

*Ecological opportunity* occurs when selection pressures on a population are relaxed due to a reduction in competition and/or predation (see Simpson 1949; Lister 1976; Harmon et al. 2008; Parent and Crespi 2009; Martin and Pfennig 2010; Yoder et al. 2010). A number of ecological and evolutionary changes, collectively referred to as *ecological release*, can occur rapidly after a population encounters ecological opportunity (MacArthur and Wilson 1967; Cox and Ricklefs 1977; Azuma 1992; Losos and DeQueiroz 1997; Bolnick et al. 2004; Yoder et al. 2010). Colonizing a new environment can be one type of ecological opportunity (Harmon et al. 2008; Parent and Crespi 2009), but ecological release can also follow the extinction of antagonists (Sepkoski 1981; Niklas et al. 1983), or the evolution of a key trait (Ehrlich and Raven 1964; Farrell 1998; Salzburger et al. 2005).

Examples of ecological opportunity and ecological release often appear in the context of adaptive radiation, especially on islands and other isolated systems (Cox and Ricklefs 1977; Schluter 1993; Losos and DeQueiroz 1997; Harmon et al. 2008; Parent and Crespi 2009; Losos 2010). However, we can also observe the consequences of ecological opportunity and ecological release in natural systems that do not exhibit adaptive radiations. For example, we can look for ecological opportunity when the evolution of a key adaptive trait has allowed a subset of species to colonize a new habitat.

Previous researchers have identified three important characteristics of ecological release following ecological opportunity. Colonists may exhibit: (1) density compensation (the new habitat can support more individuals in the absence of interspecific predation and competition [MacArthur et al. 1972; Case 1975]), (2) broadened resource use (antagonists that restrict niche width are absent [MacArthur and Wilson 1967; Lister 1976]), and (3) increased trait variation (decreased selection against individuals with extreme morphological characteristics [Bolnick et al. 2007]).

We identified ecological opportunity and tested for three characteristics of ecological release using recent lizard colonists of White Sands, New Mexico. White Sands provides a replicated natural “experiment” in which to test for rapid ecological and evolutionary change (Rosenblum 2006; Rosenblum and Harmon 2011). Three lizard species, the Eastern Fence Lizard (*Sceloporus undulatus*), the Lesser Earless Lizard (*Holbrookia maculata*), and the Little Striped Whiptail (*Aspidoscelis inornata*), colonized the recently formed gypsum sand dunes in the last 2000–6000 years (Kocurek et al. 2007). The three species have evolved white coloration in parallel, a key trait that allows them to camouflage with the stark white sands (Rosenblum 2006). The evolution from an ancestral dark pigmentation to blanched coloration has a genetic basis in all three species (Rosenblum 2005; Rosenblum et al. 2010). Because of its recent formation and novel selective environment, we hypothesize that White Sands represents a case of ecological opportunity and its resident lizards have undergone ecological release.

We looked for evidence of ecological opportunity in White Sands and examined whether lizard colonists experienced the three proposed components of ecological release. To identify ecological opportunity, we surveyed lizard communities in White Sands and ancestral dark soils habitats. We predicted that White Sands would have lower species richness and fewer potential predators and competitors. We then compared the three components of ecological release between White Sands and dark soils lizards. First, we predicted that reduced richness and abundance of predators and competitors would result in increased population sizes of the three White Sands species relative to their dark soil counterparts. Second, we predicted expanded resource use at White Sands as measured by perch choice (an important component of the lizards' ecological niche) focusing on *S. undulatus*. Third, we predicted greater morphological variation as measured by elements of body shape related to resource use in White Sands *S. undulatus* compared to conspecifics in dark soils.

## Materials and Methods

### Identifying ecological opportunity

To identify ecological opportunity, we compared species richness and relative abundance of communities in both White Sands and surrounding dark soils to determine if the number of potential antagonists (predators and competitors) of our focal species differed. We conducted field surveys on the gypsum dunes of White Sands National Monument, Otero County, New Mexico, and a typical dark soils blue-gramma grass and yucca-mesquite scrubland at Jornada Long-term Ecological Research Station, Doña Ana County, New Mexico. We measured species richness and relative abundance of the lizard communities in each habitat by performing visual encounter surveys (Campbell and Christman 1982) from May to June 2009. During each survey, two observers walked in the same direction approximately 20-m apart for 30 min. We conducted the survey procedure three times in a given habitat each day for 4 days (a total of 12 surveys per habitat). Each observer identified and counted all animals observed within a 6-m corridor. We totaled the species richness for each observer on each day and used these totals as replicates for statistical analyses. We compared species richness and relative abundance (ln transformed) of all individuals between White Sands and dark soils using *t*-tests.

We also conducted avian surveys to identify potential bird predators along a 2000-m road transect. We conducted point counts every 200 m for a total of 3 min each, counting only birds identified within approximately 100-m from the observation point. To test for differences in avian predators between White Sands and dark soils, we totaled the abundance of birds along each transect and compared the average over three surveys. We only counted birds that have been observed directly to prey on lizards or have close relatives that do so. We tested the difference in abundance of these potential bird predators using *t*-tests.

### Testing for ecological release

We tested the three different components of ecological release in White Sands lizards: density compensation, broadened resource use, and increased morphological trait variation. First, to test for density compensation, we used the results from the surveys described above to compare the abundance (total number of individuals) of each of the three focal lizard species in White Sands and dark soils. We compared abundance between White Sands and dark soils using Welch's *t*-tests (which does not require the assumption of equal variances).

Second, to test for broadened resource use, we used perch selection as a proxy for resource use of *S. undulatus*. Perch selectivity is closely related to several components of the ecological niche in lizards including diet, competition, mating behavior, and predation (Schoener 1974). To determine *S. undulatus* perch selection and to test whether perch use differed between the different habitats, we compared perch availability and perch use in White Sands and dark soils (see Rand 1964; Campbell and Christman 1982; Harmon et al. 2007). We quantified habitats from May to June in 2009 and observed lizards during their most active times of day (08:00 to 13:00, and 16:00 to 19:00). We measured perch use for 55 lizards in White Sands and 39 lizards in dark soils. We characterized the surrounding microhabitat of lizards at the exact location we first sighted them and recorded the following parameters: perch height, diameter, and distance to nearest vegetation (in meters) and canopy cover (percentage as a visual estimate to the nearest 5%). We also categorized perch surface into one of three broad categories: exposed surfaces (not associated with vegetation), yucca (often preferred by *S. undulatus*), or other non-yucca shrub/tree (association with mesquite, creosote, sage-brush, and other leafy vegetation). For each lizard, we also measured perch characteristics for a random point not associated with a lizard. The random point was found by using a random number generator to select a distance between 1 and 20-m and a direction between 0° and 360°. We performed log linear models to test whether lizards select perches proportionally to their availability, nonrandomly, or nonrandomly and differently across habitats (Heisey 1985; Manly et al. 1993). We also compared other aspects of microhabitat (perch diameter, perch height, and canopy cover) using Welch's *t*-test.

To further analyze differences in *S. undulatus* perch use across habitats, we simulated the perches that dark soil lizards might use in White Sands given their calculated selectivity indices (Manly et al. 1993). We calculated the expected perch use of a lizard with a given selectivity for a habitat type using the Heisey formula (Heisey 1985):


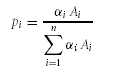


Where *n* is the number of perch types, *p_i_* is the expected probability that perch type *i* is used, α*_i_* is its availability (in White Sands), and A*_i_* is the lizard selectivity for that perch category. We calculated the expected probabilities and then drew random lizard perches (“simulated” dark soil lizards) with the same sample size as our empirical data. We then computed the Shannon diversity index (Shannon 1948) of simulated dark soils lizards 1000 times to generate a null distribution. We then compared the simulated null distribution to the Shannon diversity index of actual lizard perch use in White Sands to calculate a *P*-value.

To test whether morphological traits change in mean or variance in White Sands *S. undulatus*, we measured eight aspects of body shape following Melville et al. (2006) (shoulder–elbow, elbow–wrist, longest forelimb toe, hip–knee, knee–ankle, longest hindlimb toe length, snout vent length, head depth, head width, tail length, and pelvic width) for 20 White Sands and 18 dark soils lizards. To determine the most important axes of variation for the set of related morphological characteristics, we performed a principal components (PC) analysis on the In-transformed measurements. We compared mean body shape across habitats using multivariate analysis of variance (MANOVA) followed by post hoc analyses of covariance (ANCOVAs) on each individual variable controlling for body size. We then used Levene's test to examine whether the variance differed between White Sands and dark soils lizards for each of the first four PC. All analyses were conducted in R (R Development Core Team 2011).

## Results

### Identifying ecological opportunity

**Figure 1:**
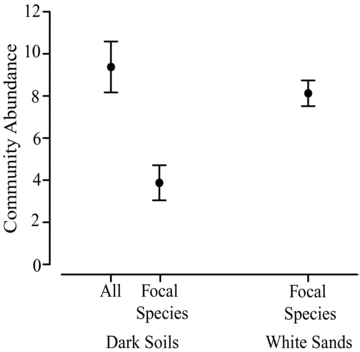
Mean lizard community abundance (number of lizards observed per transect) for all species and for the focal species in dark soils and White Sands. Circles represent the mean and error bars represent the standard error of the mean.

**Figure 2:**
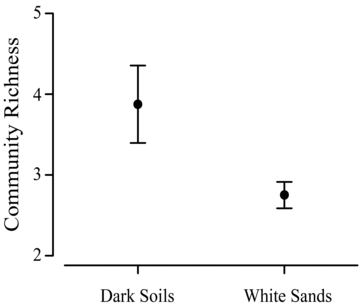
Mean lizard community richness (Shannon's *H*) in dark soils and White Sands habitat. Circles represent the mean and error bars represent the standard error of the mean.

White Sands contained fewer potential predators and competitors than the surrounding dark soils habitat and thus met a key criterion for ecological opportunity. There was no difference between the total number of lizards (of all species) between White Sands and dark soils (Fig. 1, Welch's *t*-test: *t* = 0.37, *P* = 0.72). Species richness, however, was nearly significantly higher in dark soils (Fig. 2, Welch's *t*-test: *t* = 2.22, *P* = 0.054). During our surveys, we observed three lizard species at White Sands (*A. inornata, H. maculata,* and *S. undulatus)*, compared to six lizard species in dark soils (*A. inornata, A. neomexicanus*, *A. tesselatus*, *S. undulatus, Uta stansburiana,* and *Gambelia wislizenii*). Note that although *H. maculata* does inhabit dark soils (Rosenblum 2006), we did not observe this species during our regular surveys there. We observed no additional lizard species at White Sands outside regular surveys but we documented an additional four lizard species in dark soils outside regular surveys (*Crotaphytus collaris*, *Phrynosoma cornutum*, *A. tigris,* and *S. magister*).

Total abundance of potential avian predators was higher along our dark soils transect than our White Sands transect (Welch's *t*-test: *t* = 3.25, *P* = 0.032). During our surveys, we observed the Northern mockingbird (*Mimus polyglottos*), the Common raven (*Corvus corax*), the Swainson's hawk (*Buteo swainsoni*), and various flycatchers (Tyrannidae). However, there were several key avian predators of lizards that we did not observe in either habitat during our surveys (e.g., the Loggerhead shrike [*Lanius ludovicianus*, Reid and Fulbright 1981], American kestrel [*Falco sparverius*, Craig and Trost 1979; McLaughlin and Roughgarden 1989], and the Greater roadrunner [*Geococcyx californianus*, Audsley et al. 2006]).

### Testing for ecological release

Our surveys showed evidence of density compensation in White Sands on the community level. As stated, the combined abundance of all lizards at White Sands was equivalent with that in dark soils even though species richness was significantly lower. Moreover, the combined abundance of the focal species was significantly greater in White Sands compared to dark soils (Fig. 1, Welch's t-test: *t* = –3.30, *P* = 0.002). The population sizes of each species were not significantly different between habitats when we analyzed each species separately (Welch's *t*-test: all *P*≫ 0.1)

**Figure 3:**
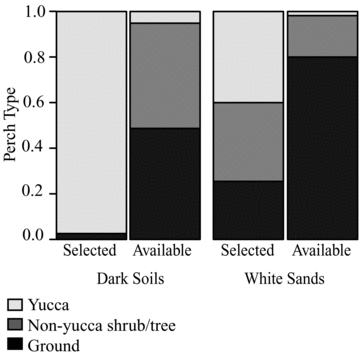
*Sceloporus undulatus* perch use compared to perch availability in dark soils and White Sands habitats. Column width corresponds to sample size in each location.

*Sceloporus undulatus* perch use was nonrandom and significantly different between White Sands and dark soils. First, lizards selected their perches nonrandomly in each habitat, that is, not proportionately to perch availability (Fig. 3, Log linear model, perch × selectivity: *F*_11,7_ = 13.7, *P*≪ 0.001). Second, lizards selected their perches differently between White Sands and dark soils (Fig. 3, Log linear model, perch × selectivity × location: *F*_11,4_ = 48.1, *P*≪ 0.001). Therefore, White Sands and dark soils *S. undulatus* differed in their perch use in a way that is not simply a reflection of perch availability in each of the two habitats.

Our simulation results demonstrated that White Sands lizards had especially diverse perch use. To control for differences in availability between the two habitats, we compared a simulation of dark soils *S. undulatus* perch use in White Sands to actual White Sands lizard perch use. We found that given their selectivities, if dark soil lizards were in White Sands they would use a more restricted range of perches than White Sands lizards (randomization test, Shannon diversity *H* = 1.08, *P* = 0.0001).

Perches selected by lizards in White Sands versus dark soils differed significantly in diameter, height, canopy cover, and distance from vegetation. White Sands lizards used perches with larger diameter (Welch's *t*-test: *t* = –3.39, *P* = 0.002), whereas dark soils lizards used perches that were higher (Welch's *t*-test: *t* = 10.5, *P* < 0.0001) and with more canopy cover (Welch's *t*-test: *t* = –2.87, *P* = 0.005). Most of the perch characteristics that we measured were more variable for White Sands lizards including perch diameter (Levene test: *F* = 8.42, *P* = 0.005), canopy cover (Levene test: *F* = 6.90, *P* = 0.01), and distance from vegetation (Levene test: *F* = 5.99, *P* = 0.02), but not perch height (Levene test: *F* = 0.38, *P* = 0.54).

Our principal component analysis of morphology showed a difference in mean trait values across habitats but no evidence of increased trait variance in White Sands lizards. We observed a difference in means across populations for morphological traits (MANOVA: Wilks’λ = 0.64, *P* = 0.04). Specifically, several fore limb and hind limb measurements differed between White Sands and dark soils *S. undulatus* (ANCOVA: habitat effect, all *P* < 0.05; body size effect, all *P* < 0.05; habitat by body size interaction, all *P* > 0.05). All other morphological variables (e.g., head and body dimensions) were related to body size but did not differ across habitats (ANCOVA: habitat effect, all *P* > 0.05). The first principal component, which corresponded primarily to body size, was actually more variable for dark soils lizards (Levene Test: *F* = 17.75, *P* < 0.01), but this was due to a bimodal distribution of sizes in the dark soils population and likely reflects the presence of more subadults in this population during the sampling period. PC2 to PC4 were not significantly different in variance between dark soils and White Sands individuals (Levene Test: all *P* > 0.1) demonstrating no evidence of increased trait variance in either habitat.

## Discussion

There is potential for ecological opportunity in many recently formed, isolated or difficult to colonize environments (MacArthur and Wilson 1967; Cox and Ricklefs 1977; Schluter 1993; Losos and DeQueiroz 1997; Harmon et al. 2008; Parent and Crespi 2009). As with many cases of ecological opportunity, the invasion of White Sands by three species of desert lizards coincided with the evolution of a key trait (Ehrlich and Raven 1964; Farrell 1998; Salzburger et al. 2005): blanched coloration that allowed substrate matching. The successful colonization of the novel White Sands habitat set the stage for ecological opportunity and release.

One of the hallmarks of ecological opportunity is reduced predation and competition, and we found fewer antagonists at White Sands. Our surveys demonstrated that the reptile community of White Sands is species poor (Fig. 2), including only three lizard species (*S. undulatus*, *H. maculata,* and *A. inornata*). Up to 35 different reptile species inhabit the dark soils habitat (see Degenhardt et al. 1996). In our dark soil surveys, we directly observed six species, including species that overlap in prey consumption with *A. inornata* (e.g., *A. neomexicanus* and *A. tesselatus* [Dixon and Medica, 1966; Scudday and Dixon 1973]) and *S. undulatus* and *H. maculata* (e.g., *U. stansburiana* [Dixon 1966]). We also observed *G. wislizenii* in our dark soils surveys, which is a known predator of all three focal species (Little and Keller 1937; Gehlbach 1956). Outside regular surveys, we recorded four additional lizard species in dark soils that either share food resources with or predate on the three focal species (i.e., *P. cornutum*, [Pianka and Parker 1975], *C. collaris* [Little and Keller 1937], *A. tigris* [Pianka 1970], and *S. magister* [Parker and Pianka 1973]). We did not observe any snakes during our surveys. In general, snakes rarely enter the central dunes of White Sands (McKeever, personal communication), but various snake species (e.g., rattlesnakes [Holycross and Mackessy 2002] and gopher snakes [Rodriguez–Robles 2002]) are common in dark soils habitat and are known to predate on the focal lizard species (Little and Keller 1937).

We also found reduced numbers of bird antagonists at White Sands. Specifically, we saw fewer avian predators (including mockingbirds, flycatchers, larger hawks, and ravens) at White Sands compared to dark soils habitat. Although the diet of these species has not been studied at White Sands per se, they may be important predators of lizards. For example, species of mockingbirds and flycatchers occasionally feed on *Anolis* lizards in the dry season in the tropics (Wunderle 1981), Swainson's hawks include desert lizards as a large part of their diet (Giovanni et al. 2007), and common ravens opportunistically forage on small reptiles (Stiehl and Trautwein 1991). Avian visual predators, such as the American kestrel, loggerhead shrike, and greater roadrunner were absent from our surveys in both dark soils and White Sands; however, we saw foraging roadrunners outside regular surveys as well as their tracks in our dark soils site. The lack of kestrels and shrikes in our observations is consistent with documentation that both have recently experienced steady population declines in North America (Hobson and Wassenaar 2001; Smallwood et al. 2009).

We found evidence that ecological opportunity in White Sands has led to two of the three main components of ecological release in its lizard inhabitants: density compensation and broadened resource use. The combined populations of all three White Sands lizard species showed density compensation, an increase in population size after colonization. We observed density compensation only at the level of the entire lizard community. Our results follow MacArthur et al. (1972) who suggested that density compensation might occur on the community scale if the total number of individuals on island populations is equivalent to that of the mainland population. At White Sands, the combined abundance of the focal species was significantly greater in White Sands compared to dark soils (Fig. 1), and the total lizard community abundance was equivalent across habitats even with lower species richness at White Sands (Fig. 2). Additionally, *H. maculata* was absent from our surveys in dark soils habitat where the three focal species are widely distributed and rarely overlap in one location (Rosenblum 2006, *personal observation*). Indeed, White Sands is one of the few locations in New Mexico with large overlapping populations of all three species. The large population sizes of the three focal species at White Sands likely reflects the reduction of predators and competitors.

Niche expansion, a key component of ecological release, was also evident in White Sands *S. undulatus* in the form of broadened perch selection. We documented expanded resource use (*i.e.*, change in niche width) by measuring perch availability compared to selectivity in this species. Perch use represents a key component of the ecological niche of sit and wait foragers such as *S. undulatus*. *Sceloporus* spp. will defend their perches from both intraspecific (Martins 1993; Haenel et al. 2003; Robertson and Rosenblum 2010) and interspecific competitors (Dunham 1980). Preferable perches could be those that offer better predator evasion (*e.g.*, Stamps 1983), access to better food resources (*e.g*., Schoener 1975), and prominent sites for display (*e.g.*, Pounds and Jackson 1983). As we predicted, in both habitats, *S. undulatus* did not select perches randomly (*i.e.*, according to their availability). Moreover, in White Sands, *S. undulatus* used more variable perches and partitioned available perches more evenly than in dark soils (Fig. 3). In both habitats, *S. undulatus* frequently perched on yuccas, which were relatively rare (see also Hager 2001). In White Sands, however, lizards selected perches that were exposed or associated with non-yucca shrubs or trees just as often as they selected yucca perches (Fig. 3). The wider variety of perch diameter, distance from vegetation, and canopy cover used by White Sands lizards was a consequence of their more diverse perch use. Further research is needed to understand the ecological mechanism for shifts in perch use across habitats. It is possible that dark soils lizards perched almost exclusively on yucca stalks high above the ground to avoid ground predators (Schoener et al. 2001; Losos et al. 2004) or as a result of resource partitioning with interspecific competitors occupying lower perches (see Schoener 1974; Losos et al. 1993). Similarly, it is possible that because White Sands *S. undulatus* forage at the base of vegetation (Dixon and Medica 1966), they prefer perches where food is nearby to those higher off the ground. A comparative analysis of the diet of lizards in both habitats would demonstrate whether perch use affects this aspect of the ecological niche.

Although density compensation and broadened resource use provide convincing evidence for ecological release in White Sands lizards, we did not observe increased trait variation. Although frequently associated with the concept of ecological release, increased trait variation was not a condition of ecological release in its original definition (MacArthur and Wilson 1967) and is rarely documented in natural populations (Schluter 2000). As such, the absence of high morphological diversity in White Sands *S. undulatus* does not negate the importance of ecological release as an evolutionary process in White Sands. Mechanistically, the lack of increased trait variation in White Sands could be explained in several ways. First, the increased niche breath that characterizes ecological release can occur without increased trait variation. For example, directional change in trait values may itself be a response to more variable resource availability. Here, we report a directional shift in *S. undulatus* limb morphology across habitats that may allow more flexibility in perch use (Irschick and Losos 1998). Similarly, the directional change toward broader heads in other White Sands lizard species (*i.e.*, *H. maculata* and *A. inornata;* Rosenblum and Harmon 2011) may enable lizards to add harder prey to their diet (Herrel et al. 2001). Second, increased trait variation may be the final step in ecological release (Yoder et al. 2010) and White Sands populations may be too young to have reached that final stage. Finally, colonization by a small number of individuals in a novel habitat could initially reduce genetic diversity (see Templeton 1979) thus reducing trait variation.

We have demonstrated that the White Sands lizards have experienced ecological opportunity in their novel habitat. White Sands is species poor, ecologically distinct from the surrounding dark soil desert, and contains only a few species with the key adaptations necessary for survival. For White Sands lizards, ecological release manifests as density compensation and broadened resource use, but not as increased morphological variation within species. We stress that the study of ecological opportunity and release should not be restricted to cases where diversification, speciation, and adaptive radiation have occurred. Likely, there are many undocumented cases of ecological release in nature that can shed light on the ecological and evolutionary changes that occur following colonization of novel habitats.
